# Dupilumab Combined With House Dust Mite Allergen Immunotherapy for Atopic Dermatitis: A Systematic Review and Meta‐Analysis

**DOI:** 10.1002/clt2.70194

**Published:** 2026-08-08

**Authors:** Jiale Lian, Ling Ren, Jing Liang, Shuping Guo

**Affiliations:** ^1^ Department of Dermatology First Hospital of Shanxi Medical University Taiyuan Shanxi China; ^2^ The First Clinical Medical College Shanxi Medical University Taiyuan Shanxi China

**Keywords:** allergen immunotherapy, atopic dermatitis, dupilumab, house dust mite

## Abstract

**Background:**

Dupilumab is effective for moderate‐to‐severe atopic dermatitis (AD), but sustained disease control after treatment discontinuation remains challenging. House dust mite allergen immunotherapy (HDM‐AIT) may provide disease‐modifying effects in sensitized patients, but the added value of combining HDM‐AIT with dupilumab remains unclear.

**Objective:**

To evaluate the efficacy and safety of dupilumab combined with HDM‐AIT versus dupilumab monotherapy in patients with AD.

**Methods:**

PubMed, Embase, Web of Science, and Cochrane Library were searched from inception to April 21, 2026. Randomized controlled trials and comparative observational studies evaluating dupilumab plus HDM‐AIT versus dupilumab alone were included. Disease severity assessed by EASI or SCORAD was pooled using standardized mean differences (SMDs), Dermatology Life Quality Index (DLQI) using mean differences, and adverse events using risk ratios. Random‐effects models were applied. The certainty of evidence was assessed using the GRADE approach.

**Results:**

Four studies involving 138 patients were included, comprising one randomized controlled trial and three comparative observational studies. Combination therapy showed no significant improvement in disease severity at 6 months (SMD = −0.02, 95% CI −0.41 to 0.36; *I* ^2^ = 0%) or 12 months (SMD = 0.53, 95% CI −1.10 to 2.16; *I* ^2^ = 93%). At 18 months, the pooled estimate suggested a possible benefit of combination therapy for disease severity, although the certainty of evidence was very low (SMD = −0.96, 95% CI −1.76 to −0.17; *I* ^2^ = 61%), although evidence was limited. At the follow‐up closest to 30 months, the effect favored combination therapy but was not significant (SMD = −1.16, 95% CI −2.56 to 0.24; *I* ^2^ = 87%). No significant differences were observed in DLQI, overall adverse events, or ocular adverse events.

**Conclusion:**

Current evidence regarding dupilumab combined with HDM‐AIT for AD remains limited and inconclusive. Although an exploratory signal of improved disease severity was observed at 18 months, the available data did not demonstrate a statistically significant difference in adverse events, and the certainty of evidence was very low for all evaluated outcomes. Further adequately powered randomized controlled trials with standardized protocols, outcome definitions, and long‐term follow‐up are needed.

**Abbreviations**
95% CI: 95% confidence intervals
AD: atopic dermatitis
AIT: allergen immunotherapy
DLQI: Dermatology Life Quality Index
EASI: Eczema Area and Severity Index
HDM‐AIT: house dust mite allergen immunotherapy
IsGA: Investigator’s Static Global Assessment
NOS: the Newcastle–Ottawa Scale
PRISMA: Preferred Reporting Items for Systematic Reviews and Meta‐Analyses
RRs: risk ratios
SCIT: subcutaneous immunotherapy
SCORAD: Scoring Atopic Dermatitis
SLIT: sublingual immunotherapy
SMD: standardized mean difference

## 1 Introduction

Atopic dermatitis (AD) is a chronic, relapsing inflammatory skin disease characterized by persistent pruritus, recurrent eczematous lesions, and substantial impairment in quality of life [1, 2]. Although topical anti‐inflammatory therapies, emollients, and systemic agents can effectively control disease activity in many patients [3], long‐term disease management remains challenging because of frequent relapse, treatment burden, and the need for sustained maintenance therapy. In recent years, biologics and selective small‐molecule targeted agents have substantially expanded the therapeutic options for moderate‐to‐severe AD [4].

Dupilumab, a monoclonal antibody targeting the interleukin‐4 receptor alpha subunit, blocks both IL‐4 and IL‐13 signaling pathways [5] and has demonstrated substantial efficacy in improving clinical symptoms in patients with AD [6]. However, dupilumab primarily suppresses type 2 inflammation and does not directly induce allergen‐specific immune tolerance [7, 8]. Therefore, disease control may still depend on continued treatment in some patients, and relapse after treatment discontinuation remains a clinical concern [9].

AD is frequently associated with atopic comorbidities and IgE‐mediated sensitization to environmental allergens [10]. Among these allergens, house dust mite is one of the most common sensitizing allergens in patients with AD and may contribute to disease exacerbation in selected individuals [11, 12]. Allergen immunotherapy (AIT), including sublingual immunotherapy (SLIT) and subcutaneous immunotherapy (SCIT), has disease‐modifying potential in IgE‐mediated allergic diseases [13] and may benefit selected sensitized patients with AD [14]. In contrast to dupilumab, house dust mite allergen immunotherapy (HDM‐AIT) aims to modify allergen‐specific immune responses by promoting immune tolerance, although its clinical effects usually develop gradually [15, 16, 17]. Therefore, combining dupilumab with HDM‐AIT may be biologically complementary: dupilumab may provide rapid control of inflammation, whereas HDM‐AIT may contribute to longer‐term immune modulation in HDM‐sensitized patients with AD.

Previous systematic reviews have evaluated AIT as a standalone or adjunctive treatment for AD [18, 19], and numerous meta‐analyses have established the efficacy and safety of dupilumab and other targeted systemic therapies for moderate‐to‐severe AD [20, 21, 22]. However, these studies did not specifically address whether adding HDM‐AIT to dupilumab provides additional benefits over dupilumab monotherapy. Several recent comparative studies have explored dupilumab combined with HDM‐AIT for AD, but the available evidence remains limited and heterogeneous in terms of study design, patient populations, AIT regimens, follow‐up durations, and outcome measures. Therefore, a focused synthesis of comparative evidence is needed to clarify the potential efficacy and safety of dupilumab combined with HDM‐AIT versus dupilumab monotherapy in patients with AD.

Therefore, this study aimed to systematically review and meta‐analyze the comparative efficacy and safety of dupilumab combined with HDM‐AIT versus dupilumab monotherapy in patients with AD. The analysis focused on disease severity, quality of life, treatment response, and adverse events at different follow‐up time points, with the aim of clarifying the potential added value and safety profile of HDM‐AIT as an adjunct to dupilumab.

## 2 Materials and Methods

### 2.1 Study Design and Registration

This systematic review and meta‐analysis was conducted in accordance with the Preferred Reporting Items for Systematic Reviews and Meta‐Analyses (PRISMA) guidelines [23]. The study protocol was registered in PROSPERO (CRD420261380622).

### 2.2 Data Sources and Search Strategy

We systematically searched PubMed, Embase, Web of Science, and the Cochrane Library from inception to April 21, 2026, to identify studies evaluating dupilumab combined with HDM‐AIT for the treatment of atopic dermatitis. The reference lists of included studies and relevant reviews were also manually screened to identify additional eligible articles.

The search strategy was independently developed by two investigators (Jiale Lian and Ling Ren) using terms related to “atopic dermatitis,” “dupilumab,” “house dust mite,” and “allergen immunotherapy.” Both controlled vocabulary terms, including MeSH and Emtree terms, and free‐text terms were used where applicable. Search terms were combined using Boolean operators (“OR” and “AND”). No restrictions were applied regarding publication year. Only English‐language articles were included. All retrieved records were imported into EndNote for duplicate removal, followed by manual verification. The complete search strategies for each database, including search dates, search fields, and applied limits, are provided in the supporting information.

### 2.3 Inclusion and Exclusion Criteria

#### 2.3.1 Inclusion Criteria

Studies were included if they met the following PICO criteria:

1. Population: patients clinically diagnosed with atopic dermatitis, regardless of age or sex. Given the focus of this review, patients were required to have evidence of house dust mite sensitization when such information was reported.
2. Intervention: combination therapy with dupilumab and HDM‐AIT, including subcutaneous immunotherapy or sublingual immunotherapy.
3. Comparator: dupilumab monotherapy. Concomitant background treatments, such as topical corticosteroids, topical calcineurin inhibitors, emollients, or antihistamines, were allowed if they were permitted in both groups.
4. Outcomes: studies reporting at least one relevant efficacy or safety outcome, including disease severity scores, quality‐of‐life scores, treatment response, or adverse events, with data available for quantitative or qualitative synthesis.
5. Study design: peer‐reviewed randomized controlled trials or comparative observational studies, including prospective or retrospective cohort studies and case‐control studies.

#### 2.3.2 Exclusion Criteria

Studies were excluded if they met any of the following criteria:

1. Studies without a comparator group receiving dupilumab monotherapy;
2. Reviews, conference abstracts, comments, letters, case reports, case series, editorials, or expert opinions;
3. Single‐arm studies or studies evaluating dupilumab combined with HDM‐AIT without a relevant comparative group;
4. Studies in which the full text or relevant outcome data could not be retrieved or obtained after reasonable attempts;
5. Non‐English publications.

### 2.4 Study Selection

All retrieved records were imported into EndNote for duplicate removal, followed by manual verification. After duplicates were removed, two investigators (Jiale Lian and Ling Ren) independently screened the titles and abstracts of all records according to the predefined eligibility criteria. Studies that were clearly irrelevant were excluded at this stage. The full texts of potentially eligible articles were then retrieved and independently assessed by the same two investigators. Any disagreements during title/abstract screening or full‐text assessment were resolved through discussion and consensus; when necessary, a third investigator (Shuping Guo) was consulted.

### 2.5 Data Extraction

Data were extracted using a predesigned standardized form in Microsoft Excel. Two investigators (Jiale Lian and Ling Ren) independently extracted data from each included study and cross‐checked the results. Extracted information included the first author, publication year, country, study design, sample size, patient characteristics, diagnostic criteria, evidence of house dust mite sensitization, intervention and comparator regimens, follow‐up duration, outcome measures, and adverse events.

For efficacy outcomes, data were extracted at predefined follow‐up time points, including 6, 12, 18 months, and the longest available follow‐up closest to 30 months. When both endpoint values and changes from baseline were available, endpoint values were preferentially extracted to maintain consistency across studies. Disease severity outcomes included EASI and SCORAD scores, and quality‐of‐life outcomes included DLQI or CDLQI where applicable. For dichotomous outcomes, the number of events and total number of participants in each group were extracted.

When numerical data were not directly available from the text or tables, we attempted to contact the corresponding authors for additional information. If no response was received, data were extracted from published figures using WebPlotDigitizer [24]. Figure extraction was performed independently by two investigators, and extracted values were compared to minimize measurement error. For continuous outcomes reported as medians with interquartile ranges, means and standard deviations were estimated using established conversion methods. Any discrepancies during data extraction were resolved through discussion and consensus; when necessary, a third investigator was consulted.

### 2.6 Quality Assessment

To ensure methodological rigor, different assessment tools were used to evaluate the quality of studies with different designs. Observational cohort studies were assessed using the Newcastle–Ottawa Scale (NOS), which evaluates three domains: selection, comparability, and outcome/exposure.

For randomized controlled trials, methodological quality was assessed using the RoB 2 tool, which includes five domains: the randomization process, deviations from intended interventions, missing outcome data, measurement of the outcome, and selection of the reported result. Each signaling question within these domains was judged as “Yes,” “Probably yes,” “No,” “Probably no,” or “Not applicable.” The overall risk of bias for each domain was then classified as “low risk,” “some concerns,” or “high risk.”

### 2.7 Statistical Analysis

Data analyses were performed using Review Manager version 5. Disease severity scores and Dermatology Life Quality Index (DLQI) scores were quantitatively synthesized using means and standard deviations. For continuous outcomes reported as medians with interquartile ranges, means and standard deviations were estimated using established conversion methods [25]. For disease severity assessment, EASI and SCORAD were used as different scoring systems. All scales were aligned so that lower scores indicated better outcomes. Therefore, the standardized mean difference (SMD) with 95% confidence intervals (CIs) was used as the effect measure. For dichotomous outcomes, such as the occurrence of adverse events, risk ratios (RRs) were calculated as the effect measure. Considering the potential clinical and methodological heterogeneity across studies in terms of study populations, interventions, and outcome definitions, all analyses were performed using a random‐effects model. Between‐study heterogeneity was assessed using the *I* ^2^ statistic, with *I* ^2^ values of 25%, 50%, and 75% indicating low, moderate, and high heterogeneity, respectively. When feasible, sensitivity analyses were performed to explore potential sources of heterogeneity and assess the robustness of the results. A *p* value < 0.05 was considered statistically significant.

Evidence certainty for the main outcomes was assessed using the GRADE framework in GRADEpro GDT. Judgments were based on risk of bias, inconsistency, indirectness, imprecision, and publication bias [26]. For outcomes including both randomized and observational evidence, certainty was judged based on the overall body of evidence [27].

## 3 Results

### 3.1 Included Studies and Their Characteristics

**FIGURE 1:**
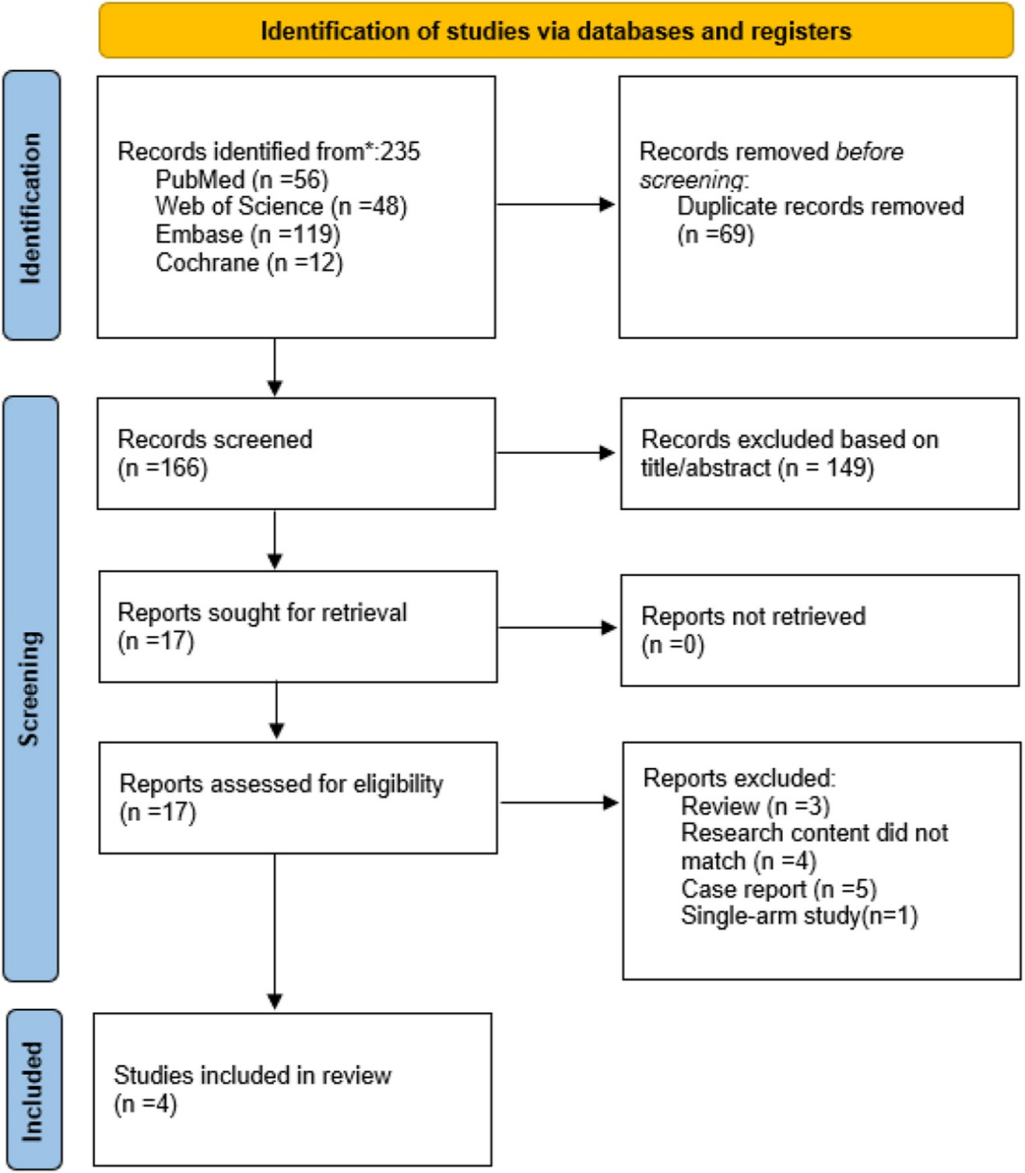
PRISMA flow diagram.

A total of 235 records were identified through searches of PubMed, Embase, Web of Science, and the Cochrane Library. After 69 duplicate records were removed, 166 records remained for screening. Following title and abstract screening, 149 records were excluded, and 17 full‐text articles were assessed for eligibility. According to the predefined eligibility criteria, 13 articles were further excluded for the following reasons: reviews (*n* = 3), irrelevant research content (*n* = 4), case reports (*n* = 5), and single‐arm study (*n* = 1). Ultimately, four studies [28, 29, 30, 31], including one randomized controlled trial and three observational cohort studies, were included in the meta‐analysis. The study selection flow diagram, characteristics of the included studies, and baseline characteristics are presented in Figure 1, Tables 1 and 2, respectively. All included studies evaluated HDM‐AIT.

**TABLE 1:** Characteristics of the included studies.

Study, year	Country	Design	Treatment groups	Sample size	Intervention	Outcomes	Follow‐up
Bogacz‐Piaseczyńska (2024) [28]	Poland	RCT	Dupilumab + SLIT‐HDM	18	ACARIZAX, one tablet daily +a single initial dose of 600 mg of dupilumab and subsequent doses of 300 mg every 2 weeks.	EASI, %BSA, IsGA, DLQI, TMS,Immunological Parameters, tIgE, sIgE to D. pter, sIgE to D. far, IgG4 against D. pter, IgG4 against D. far, safety	12 months
			Dupilumab	14	A single initial dose of 600 mg of dupilumab and subsequent doses of 300 mg every 2 weeks.		
Kim (2024) [29]	Korea	Comparative cohort study	Dupilumab + SCIT	14	SCIT with an HDM allergen extract composed of a 1:1 mixture of d1 and d2 extracts, adsorbed to tyrosine + a single initial dose of 600 mg of dupilumab and subsequent doses of 300 mg every 2 weeks.	EASI, tIgE, d1 sIgE, d2 sIgE, d1 specific IgG4, d2 specific IgG4, safety	6, 12, 18, 24, 36 months
			Dupilumab	14	An initial 600‐mg injection, followed by 300 mg biweekly.		
Liu (2025) [30]	China	Comparative cohort study	Dupilumab + SCIT	16	Inject AlutardSQ (ALK‐Abello A/S, Denmark) once a week + an initial dose of 600 mg (> 18 years old) or 300 mg (< 18 years old), and thereafter every 2 weeks (> 18 years old) or every 4 weeks (< 18 years old) with 300 mg each injection.	SCORAD, POEM,Itch‐NRS, ADCT, DLQI, CSMS, tIgE, Der p ‐sIgE, Der f‐sIgE,sIgG 4, IL‐4, IL‐13, IL‐17A, CCL17, IL‐10, IFN‐γ, PBMC, safety	6 months
			Dupilumab	16	An initial dose of 600 mg (> 18 years old) or 300 mg (< 18 years old), and thereafter every 2 weeks (> 18 years old) or every 4 weeks (< 18 years old) with 300 mg each injection.		
Ye (2025) [31]	China	Comparative cohort study	Dupilumab + SCIT	22	Dupilumab was given for 10–12 months, with SCIT added at month 6 and titrated to 5000 TU/mL.	SCORAD, CSMS,VAS, Itch score, Sleep quality score, CDLQI,tIgE, sIgE	6, 12, 18, 30 months
			Dupilumab	24	Patients aged 6 months to 5 years received weight‐based dosing, 200 mg every 4 weeks (Q4W) for < 15 kg or 300 mg Q4W for 15–30 kg, without loading doses. For patientsaged 6–17 years, an initial weight‐based loading dose (400 mg or 600 mg) was administered		

**TABLE 2:** Baseline characteristics.

Study, year	Bogacz‐Piaseczyńska (2024) [28]		Kim (2024) [29]		Liu (2025) [30]		Ye (2025) [31]	
Group	Dupilumab + SLIT‐HDM	Dupilumab	Dupilumab + SCIT	Dupilumab	Dupilumab + SCIT	Dupilumab	Dupilumab + SCIT	Dupilumab
Age (year) (mean ± SD)	19.2 ± 7.4	23.1 ± 5.1	26.9 ± 7.94	32.2 ± 8.40	15.65 ± 13.12	9.68 ± 8.73	10.83 ± 5.19	9.50 ± 4.07
Gender, women (%)	42	41	28.6	35.7	21.1	47.37	40.9	41.7
Duration of AD (years) (mean ± SD)	14.9 ± 6.2	14.3 ± 2.9	NA	NA	6.13 ± 5.02	5.21 ± 5.14	9.18 ± 3.65	6.46 ± 2.95
EASI (mean ± SD)	50 ± 8.8	45 ± 6.2	32.1 ± 7.79	32.8 ± 8.02	NA	NA	NA	NA
SCORAD (mean ± SD)	NA	NA	NA	NA	46.46 ± 18.09	55.38 ± 19.23	63.46 ± 16.59	60.25 ± 18.45
Itch score/Itch‐NRS	NA	NA	NA	NA	6.32 ± 2.63	7.84 ± 1.34	76.67 ± 11.11	70.00 ± 14.81
tIgE, KU/L,IU/L	8500 ± 4791	11,460 ± 5322	3856.7 ± 1910.9	4463.4 ± 868.6	1386.00 ± 1553.00	1445.00 ± 2005.00	2325.05 ± 1586.04	1812.92 ± 897.85
sIgE to D. pter	19.3 ± 5.3	8.9 ± 4.2	68.8 ± 33.0	77.5 ± 30.0	58.79 ± 38.03	40.95 ± 38.93	NA	NA
sIgE to D. far	9.4 ± 1.7	10.2 ± 3.3	79.4 ± 30.7	85.3 ± 27.1	70.11 ± 36.31	47.44 ± 42.61	NA	NA
sIgG4 to D. pter	NA	NA	0.71 ± 0.65	0.93 ± 1.78	NA	NA	NA	NA
sIgG4 to D. far	NA	NA	0.84 ± 0.78	1.20 ± 2.61	NA	NA	NA	NA
DLQI (mean ± SD)	12.6 ± 1.8	10.9 ± 2.7	NA	NA	9.74 ± 4.61	12.11 ± 8.33	14.59 ± 2.40	13.88 ± 2.79

### 3.2 Quality Assessment

We assessed the methodological quality of the three observational studies using the Newcastle–Ottawa Scale (NOS) [32] (See Supporting Information S1), and evaluated the randomized controlled trial using the RoB 2 tool [33] (See Supporting Information S1).

### 3.3 Disease Severity Scores at Each Follow‐Up Time Point

Four studies involving a total of 138 participants reported changes in disease severity. Standardized mean differences were used in this study to meta‐analyze the pooled effects of dupilumab combined with HDM‐AIT on disease severity in patients with AD, as assessed by EASI or SCORAD, at 6, 12, 18, and ≥ 30 months.

**FIGURE 2:**
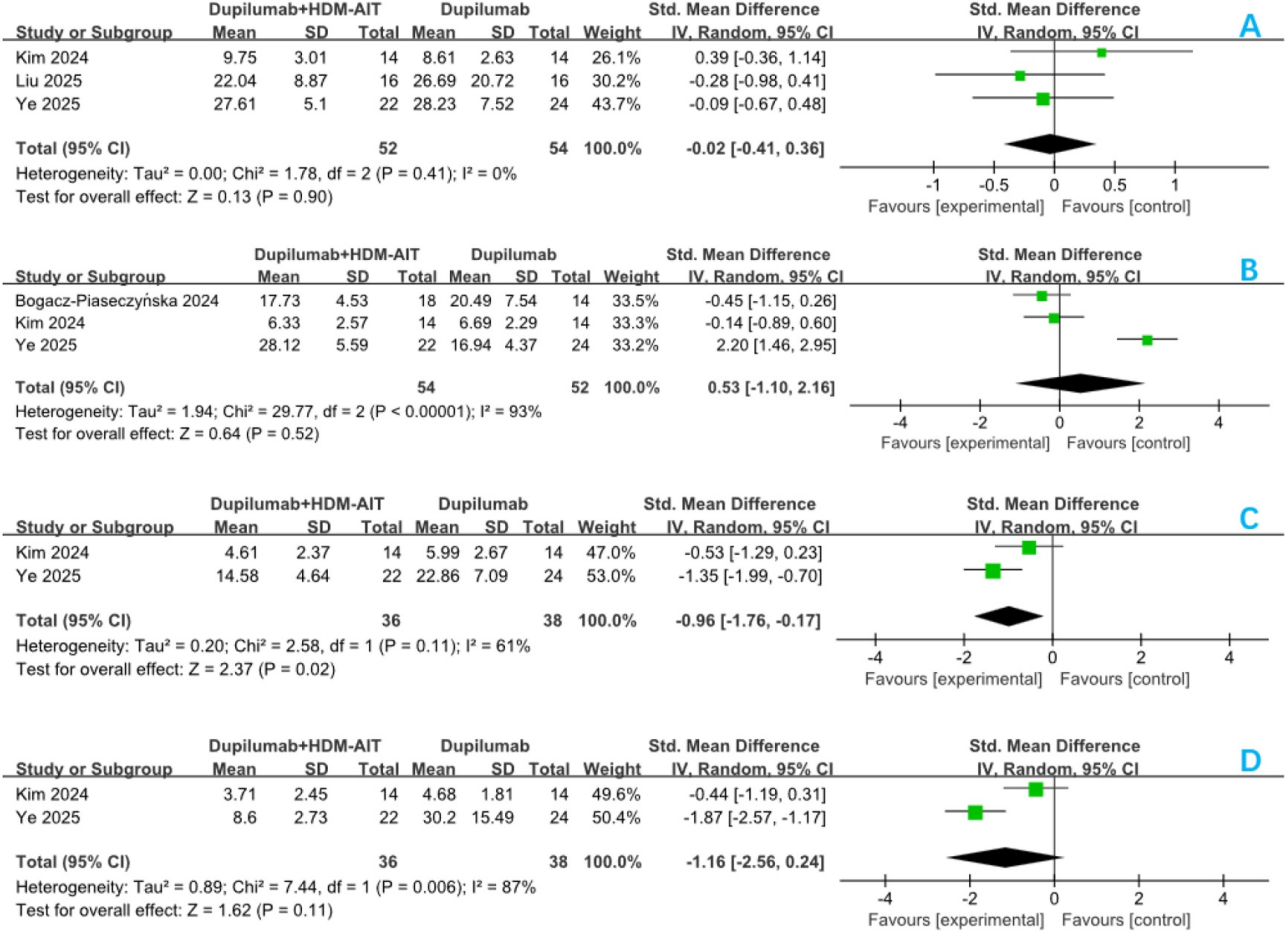
(A) Disease severity at 6 months, (B) disease severity at 12 months, (C) disease severity at 18 months, and (D) disease severity at ≥ 30 months.

The results showed that, at 6 months, there was no significant difference between combination therapy and dupilumab monotherapy (SMD = −0.02, 95% CI: −0.41 to 0.36, *I* ^2^ = 0%, *p* = 0.90; Figure 2A). At 12 months, no statistically significant difference was observed, with substantial heterogeneity across studies (SMD = 0.53, 95% CI: −1.10 to 2.16, *I* ^2^ = 93%, *p* = 0.52; Figure 2B). At 18 months, the pooled estimate favored combination therapy for disease severity (SMD = −0.96, 95% CI: −1.76 to −0.17, *I* ^2^ = 61%, *p* = 0.02; Figure 2C); However, this finding should be interpreted with caution because it was based on only two studies and very low certainty evidence. During long‐term follow‐up beyond 30 months, combination therapy showed a trend toward greater improvement, although the difference did not reach statistical significance (SMD = −1.16, 95% CI: −2.56 to 0.24, *I* ^2^ = 87%, *p* = 0.11; Figure 2D).

Overall, dupilumab combined with HDM‐AIT showed no clear advantage at 6 or 12 months. Although an exploratory benefit was observed at 18 months, this finding should be interpreted cautiously because of the small number of studies and between‐study heterogeneity.

### 3.4 Dermatology Life Quality Index (DLQI) Score

**FIGURE 3:**
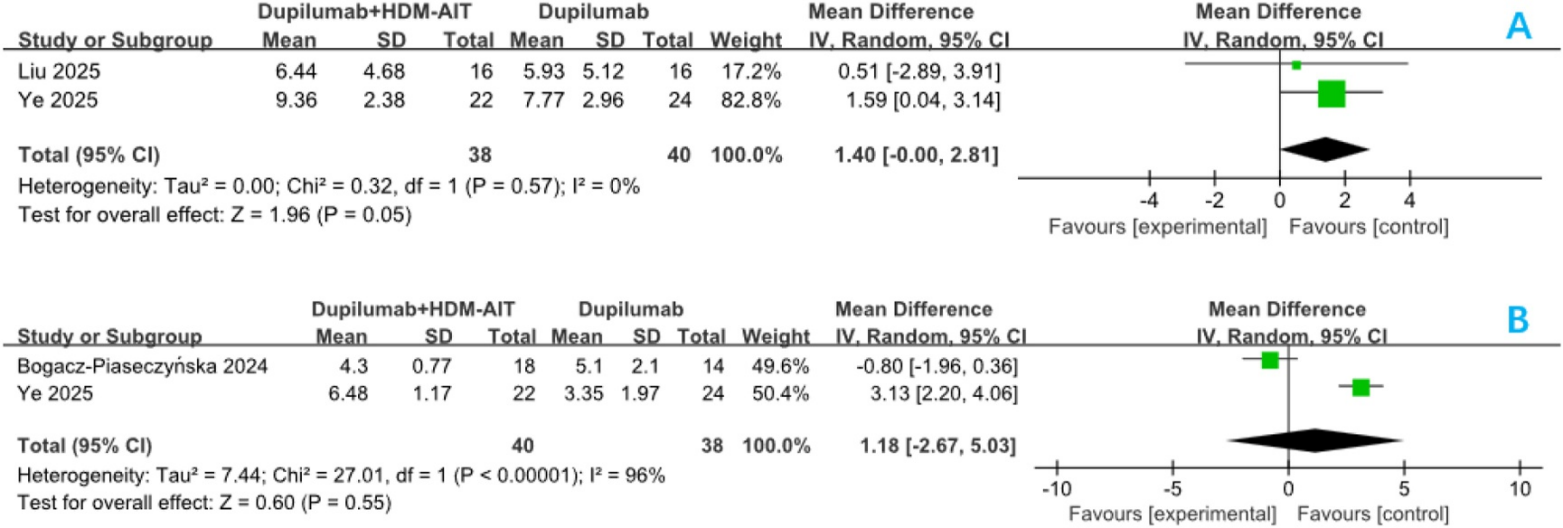
(A) DLQI at 6 months and (B) DLQI at 12 months.

Two studies reported DLQI scores at 6 and 12 months. Because lower DLQI scores indicate better quality of life, positive MD values favored dupilumab monotherapy, whereas negative MD values favored dupilumab combined with HDM‐AIT. At 6 months, no statistically significant difference in DLQI was observed between the combination therapy and dupilumab monotherapy groups, although the point estimate favored dupilumab monotherapy (MD = 1.40, 95% CI: 0.00 to 2.81, *I* ^2^ = 0%, *p* = 0.05; Figure 3A). At 12 months, the difference between the two groups remained non‐significant, with substantial heterogeneity (MD = 1.18, 95% CI: −2.67 to 5.03, *I* ^2^ = 96%, *p* = 0.55; Figure 3B).

### 3.5 Safety

All four studies assessed the safety of dupilumab combined with HDM‐AIT and dupilumab monotherapy based on the occurrence of adverse events (Table 3). The main safety concerns included further worsening of skin lesions during treatment, local injection‐site reactions associated with HDM‐AIT, and ocular symptoms related to dupilumab use. Because definitions of overall adverse events varied across studies and included both HDM‐AIT‐related local reactions and dupilumab‐related events, the pooled analysis of any adverse events was considered exploratory.

**TABLE 3:** Adverse events.

Study, year	Bogacz‐Piaseczyńska (2024) [28]		Kim (2024) [29]		Liu (2025) [30]		Ye (2025) [31]	
	Dupilumab + SLIT‐HDM	Dupilumab	Dupilumab + SCIT	Dupilumab	Dupilumab + SCIT	Dupilumab	Dupilumab + SCIT	Dupilumab
Adverse events	11/18	2/14	13/14	14/14	8/16	2/16	4/22	8/24
Injection site reactions	8 (44.4%)	0 (0.0%)	2 (14.3%)	0 (0.0%)	2 (10.53%)	0 (0.0%)	NA	NA
Aggravated skin lesions or pruritus	NA	NA	NA	NA	5 (26.3%)	0 (0.0%)	NA	3 (12.5%)
Eye symptoms	0 (0.0%)	2 (14.3%)	3 (21.4%)	4 (28.6%)	1 (5.3%)	2 (10.5%)	4 (18.2%)	5 (20.8%)
Rash and/or episode of urticaria	3 (16.7%)	0 (0.0%)	NA	NA	NA	NA	NA	NA
Dupilumab‐associated head and neck dermatitis	NA	NA	3 (21.4%)	7 (50.0%)	NA	NA	NA	NA
Upper respiratory tract infections	NA	NA	2 (14.3%)	0 (0.0%)	NA	NA	NA	NA
Herpes simplex infections	NA	NA	3 (21.4%)	3 (21.4%)	NA	NA	NA	NA

#### 3.5.1 Occurrence of Any Adverse Events

**FIGURE 4:**
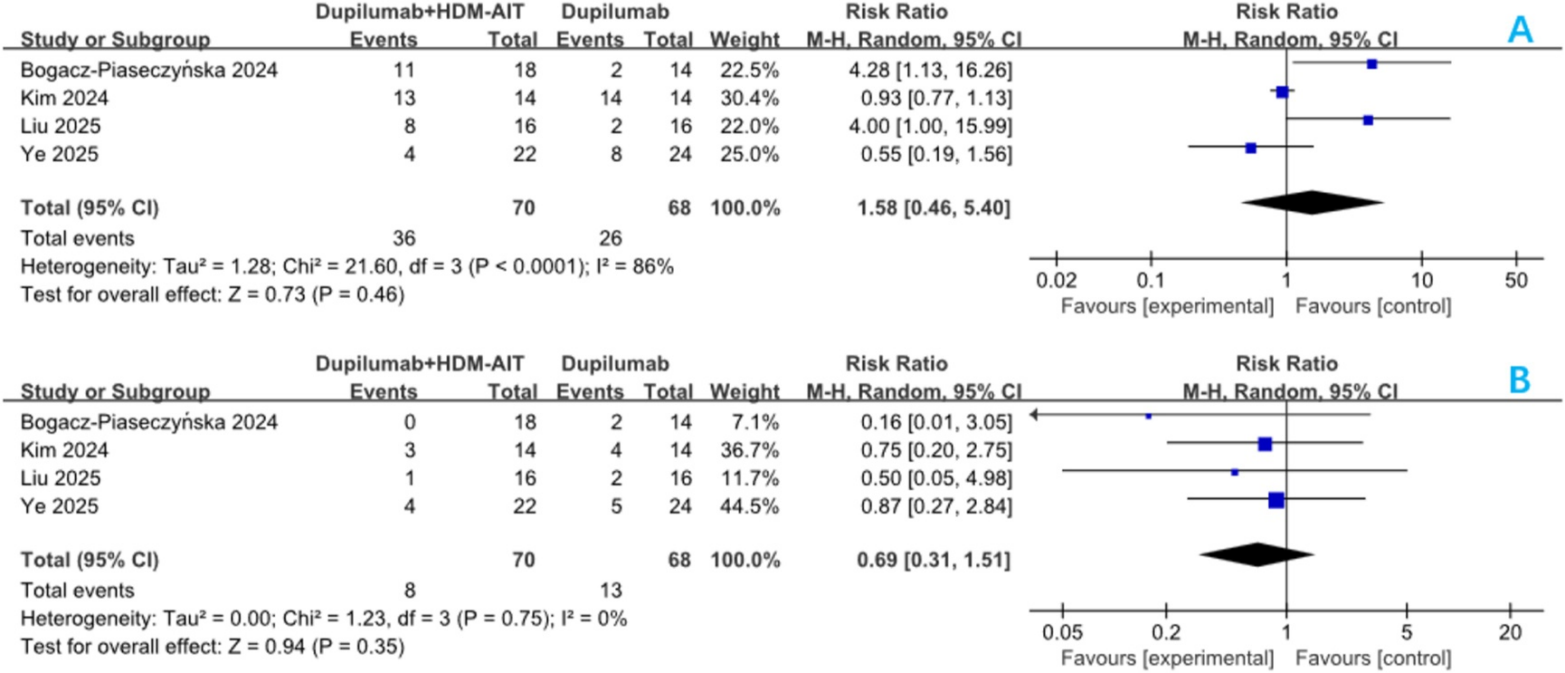
(A) Any adverse events and (B) ocular adverse events.

A meta‐analysis of all adverse events reported in the four included studies showed no significant difference in the occurrence of adverse events between the two groups (RR = 1.58, 95% CI: 0.46 to 5.40, *I* ^2^ = 86%, *p* = 0.46; Figure 4A).

#### 3.5.2 Occurrence of Ocular Adverse Events

All four studies reported ocular adverse events, including dry eye, ocular itching, tearing, or conjunctivitis. The pooled analysis showed no statistically significant difference in ocular adverse events between dupilumab combined with HDM‐AIT and dupilumab monotherapy (RR = 0.69, 95% CI: 0.31 to 1.51, *I* ^2^ = 0%, *P* = 0.35; Figure 4B). Given the limited number of events and the wide confidence interval, this finding should be interpreted cautiously.

#### 3.5.3 Other Adverse Events

Other reported adverse events included injection‐site reactions, aggravated skin lesions or pruritus, rash or urticaria, dupilumab‐associated head and neck dermatitis, upper respiratory tract infections, and herpes simplex infections. Injection‐site reactions were reported only in the combination therapy groups, which was consistent with expected HDM‐AIT‐related local reactions. Across the included studies, most reported adverse events were mild to moderate, and no life‐threatening systemic reactions or fatal events were reported.

In Bogacz‐Piaseczyńska et al. [28], rash or urticaria was reported in 3 of 18 patients in the combination therapy group and in 0 of 14 patients in the dupilumab monotherapy group. Kim et al. [29] reported dupilumab‐associated head and neck dermatitis in 3 of 14 patients in the combination therapy group and 7 of 14 patients in the dupilumab monotherapy group. Upper respiratory tract infections were reported in 2 of 14 patients in the combination therapy group and in none of the patients receiving dupilumab monotherapy. Herpes simplex infections occurred in 3 of 14 patients in each group. Liu et al. [30] reported aggravated skin lesions or pruritus in 5 of 16 patients in the combination therapy group and in none of the patients receiving dupilumab monotherapy. Ye et al. [31] reported aggravated skin lesions or pruritus in 3 of 24 patients in the dupilumab monotherapy group, whereas such events were not reported in the combination therapy group.

### 3.6 Treatment Response

Two studies reported treatment response outcomes using different definitions. Bogacz‐Piaseczyńska et al. [28] reported a higher proportion of patients achieving IsGA improvement in the combination group compared with dupilumab monotherapy. Liu et al. [30] reported SCORAD50 response rates, which were comparable between groups. Due to heterogeneity in definitions, a quantitative synthesis was not performed.

### 3.7 Sensitivity Analysis

Where applicable, sensitivity analyses were performed using a stepwise leave‐one‐out approach to explore potential sources of heterogeneity and assess the robustness of the results (see supporting information). Sensitivity analysis showed that the heterogeneity in the 12‐month outcome was primarily driven by the study by Ye et al. [31] After exclusion, heterogeneity decreased significantly (*I* ^2^ = 0%), while the overall effect remained non‐significant. This may be related to differences in patient population (pediatric cohort), treatment sequencing, and dupilumab discontinuation strategy in that study.

### 3.8 Certainty of Evidence

Evidence certainty for the main outcomes is summarized in the supporting information. All outcomes were rated as very low certainty due to imprecision, heterogeneity, and reliance on a small body of predominantly observational evidence. Publication bias could not be assessed because fewer than 10 studies were available for each outcome.

## 4 Discussion

This review synthesized the available comparative evidence on dupilumab combined with HDM‐AIT versus dupilumab monotherapy in patients with AD. Overall, combination therapy did not show a clear advantage in improving disease severity at 6 or 12 months, whereas an exploratory benefit was observed at 18 months. At the follow‐up closest to 30 months, the effect favored combination therapy, but the difference was not statistically significant. No significant differences were observed in DLQI or adverse events between the two groups. The results did not show a consistent benefit across all time points, but they raised the hypothesis of a possible delayed treatment effect.

The time‐dependent pattern observed in this meta‐analysis may partly be interpreted in light of the distinct mechanisms of dupilumab and HDM‐AIT, although this explanation remains hypothesis‐generating. Dupilumab rapidly suppresses type 2 inflammation by blocking IL‐4 and IL‐13 signaling pathways [34], which may account for early clinical improvement in both treatment groups. In this context, a potential ceiling effect of dupilumab may have limited the ability of the included studies to detect any incremental short‐term benefit of HDM‐AIT [35]. However, dupilumab primarily suppresses type 2 inflammation and is not considered to directly induce allergen‐specific immune tolerance. In contrast, HDM‐AIT has a slower onset of action and may gradually modulate allergen‐specific immune responses through mechanisms such as regulatory T‐cell induction and IgG4 production [36, 37]. Therefore, the absence of a clear additional benefit at early follow‐up may partly reflect the dominant short‐term anti‐inflammatory effect of dupilumab, whereas the exploratory signal observed at 18 months may be consistent with, but does not confirm, a delayed immunomodulatory effect of HDM‐AIT.

HDM‐AIT generally has a slower onset of action and may not induce clinically evident immune tolerance within a short period. Whether HDM‐AIT can help maintain disease control after dupilumab dose reduction or discontinuation remains an important but unproven hypothesis [38, 39]. This possibility is supported by the study by Ye et al. [31], in which multiple clinical parameters worsened after dupilumab discontinuation in the monotherapy group, whereas improvements were maintained in the combination therapy group, with the between‐group difference gradually increasing during subsequent follow‐up. However, because this finding was derived from a single study, it should be interpreted cautiously. Experimental studies suggest that SCIT may modulate Th2‐type inflammation and induce blocking antibodies; however, current clinical evidence does not support sustained immunologic modulation or a definitive disease‐modifying effect in patients with AD [40].

Regarding safety, the overall incidence of adverse events was not significantly increased with combination therapy, and local injection‐site reactions were expected HDM‐AIT‐related events [41, 42]. No statistically significant increase in ocular adverse events was observed with combination therapy. Whether HDM‐AIT may reduce dupilumab‐associated ocular adverse events remains uncertain because of the limited number of events and small sample size. Across the four included studies, the reported adverse events were generally mild, and no life‐threatening events were observed; however, the small sample size limits firm conclusions regarding safety [43, 44]. In addition, Ye et al. [31] reported one patient who experienced further worsening of skin lesions during dupilumab treatment and was subsequently switched to upadacitinib therapy.

This study has several strengths. First, it specifically focused on comparative evidence evaluating dupilumab combined with HDM‐AIT versus dupilumab monotherapy, thereby addressing a clinically relevant question that has not been adequately summarized in previous reviews. Second, we performed time‐stratified analyses, which allowed the treatment effects to be assessed across different follow‐up durations and provided a framework for examining whether the potential contribution of HDM‐AIT may vary over time. Third, both efficacy and safety outcomes were evaluated, including disease severity, quality of life, treatment response, overall adverse events, and ocular adverse events. Finally, by limiting the interpretation to HDM‐AIT, this review provides a more precise synthesis of the currently available evidence and highlights key uncertainties that should be addressed in future studies.

Differences in clinical characteristics across the included studies should be considered when interpreting the results for each outcome. The study populations were not fully comparable. For example, the randomized SLIT‐HDM trial enrolled adults with severe AD without respiratory allergic comorbidities, whereas the long‐term SCIT study focused on children and adolescents with moderate‐to‐severe AD and type 2 inflammation‐related allergic comorbidities. The other real‐world studies included adults with severe refractory AD or broader populations of HDM‐sensitized patients with AD [28, 29, 30, 31]. Differences in age, baseline disease severity, and allergic comorbidities may have affected patients' responses to dupilumab and HDM‐AIT.

The HDM‐AIT protocols differed substantially across studies. The randomized trial [28] used a standardized SLIT‐HDM tablet containing D. pteronyssinus and D. farinae extracts at a fixed daily dose for 12 months, whereas the observational studies used SCIT with different HDM extracts and build‐up schedules. In Kim et al. [29], dupilumab was added to ongoing SCIT only after an inadequate response to 6 months of SCIT, while Liu et al. [30]. evaluated concurrent SCIT and dupilumab for 6 months. In contrast, Ye et al. [31] used a pediatric long‐term strategy in which dupilumab was discontinued after 10–12 months, while SCIT was initiated at month 6 and continued through 30 months of follow‐up. Differences in HDM‐AIT route, treatment timing, and duration are clinically relevant and may partly explain differences in effect estimates across follow‐up time points. Continuous combination therapy may mainly reflect short‐term additive anti‐inflammatory effects, while discontinuation‐ or delayed initiation–based studies may reflect maintenance of disease control after biologic withdrawal rather than true additive efficacy.

Differences in outcome assessment may also have contributed to heterogeneity. Disease severity was mainly assessed using EASI or SCORAD, two instruments that evaluate related but not identical dimensions of AD severity [28, 29, 30, 31]. Although standardized mean differences were used to account for the different scales, EASI and SCORAD may still not be fully interchangeable. In addition, the composite outcome of “any adverse events” included heterogeneous events, such as local HDM‐AIT‐related reactions, ocular symptoms, infections, and head‐and‐neck dermatitis [28, 29, 30, 31]. With only four available studies, we were unable to perform reliable subgroup analyses by age group, HDM‐AIT route, treatment sequence, or outcome scale. Accordingly, we explored these sources of heterogeneity only in a qualitative manner.

Several limitations should be acknowledged. First, the number of included studies was small, and most were observational in design, which may introduce selection bias, confounding, and other methodological limitations. Second, the total sample size was limited, and several analyses included only a small number of studies, reducing the statistical power and precision of the pooled estimates. Third, substantial heterogeneity was observed in some outcomes, particularly at 12 months and in overall adverse events, which may be explained by differences in study design, patient populations, baseline disease severity, HDM‐AIT protocols, dupilumab treatment duration, discontinuation strategies, follow‐up durations, and outcome definitions. Fourth, outcome definitions varied across studies, especially for treatment response and adverse events, limiting the feasibility of quantitative synthesis for some clinically relevant outcomes. Fifth, only English‐language publications were included, which may have introduced language bias. Finally, all evaluated outcomes were rated as very low certainty in the GRADE assessment. This limits the strength of any conclusions drawn from the current evidence, but the findings may still help inform the design of future trials.

## 5 Conclusion

In conclusion, comparative data on dupilumab combined with HDM‐AIT in AD remain limited. The 18‐month analysis suggested a possible signal favoring combination therapy in disease severity, but this should be regarded as hypothesis‐generating given the very low certainty of evidence, and current data do not allow conclusions regarding sustained long‐term efficacy or safety. This review highlights the existing evidence gap in this field and provides a basis for future research. Prospective randomized controlled trials with adequate power, standardized HDM‐AIT protocols and dupilumab regimens, clearly defined treatment strategies, uniform outcome measures, and longer follow‐up are required.

## Author Contributions

**Jiale Lian:** conceptualization, methodology, software, data curation, visualization, writing – original draft. **Ling Ren:** methodology, software. **Jing Liang:** data curation, software. **Shuping Guo:** writing – review and editing, supervision, formal analysis.

## Funding

The authors have nothing to report.

## Ethics Statement

This study was a systematic review and meta‐analysis based on previously published studies. Therefore, ethical approval and informed consent were not required.

## Conflicts of Interest

The authors declare no conflicts of interest.

## Supporting information


Supporting Information S1

## Data Availability

The data used in this systematic review and meta‐analysis were derived from published articles. All extracted data are available within the article and its Supporting Information S1. Additional data supporting the findings of this study are available from the corresponding author upon reasonable request.

## References

[1] S. Meledathu , M. P. Naidu , and P. M. Brunner , “Update on Atopic Dermatitis,” Journal of Allergy and Clinical Immunology 155, no. 4 (2025): 1124–1132, 10.1016/j.jaci.2025.01.013. 39855361

[2] G. Biazus Soares , T. Hashimoto , and G. Yosipovitch , “Atopic Dermatitis Itch: Scratching for an Explanation,” Journal of Investigative Dermatology 144, no. 5 (2024): 978–988, 10.1016/j.jid.2023.10.048. 38363270

[3] A. de Benedetto , M. Boguniewicz , P. Y. Ong , D. K. Chu , and L. C. Schneider , “Atopic Dermatitis (Eczema) Guidelines 2023: Highlights,” Journal of Allergy and Clinical Immunology: In Practice 12, no. 11 (2024): 2955–2965, 10.1016/j.jaip.2024.08.052. 39251015

[4] S. Butala , L. Castelo‐Soccio , R. Seshadri , et al., “Biologic Versus Small Molecule Therapy for Treating Moderate to Severe Atopic Dermatitis: Clinical Considerations,” Journal of Allergy and Clinical Immunology: In Practice 11, no. 5 (2023): 1361–1373, 10.1016/j.jaip.2023.03.011. 36948491 PMC10164714

[5] B. Cabanillas , “Dupilumab for Atopic Dermatitis‐From Clinical Trials to Molecular and Cellular Mechanisms,” Dermatitis 34, no. 1 (2023): 21–28, 10.1089/DERM.0000000000000905. 36705657

[6] A. S. Halling , N. Loft , J. I. Silverberg , E. Guttman‐Yassky , and J. P. Thyssen , “Real‐World Evidence of Dupilumab Efficacy and Risk of Adverse Events: A Systematic Review and Meta‐Analysis,” Journal of the American Academy of Dermatology 84, no. 1 (2021): 139–147, 10.1016/j.jaad.2020.08.051. 32822798

[7] A. Głobińska , T. Boonpiyathad , P. Satitsuksanoa , et al., “Mechanisms of Allergen‐Specific Immunotherapy: Diverse Mechanisms of Immune Tolerance to Allergens,” Annals of Allergy, Asthma, & Immunology 121, no. 3 (2018): 306–312, 10.1016/j.anai.2018.06.026. 29966703

[8] S. B. Sindher , K. C. Nadeau , R. S. Chinthrajah , et al., “Efficacy and Safety of Dupilumab in Children With Peanut Allergy: A Multicenter, Open‐Label, Phase II Study,” Allergy 80, no. 1 (2025): 227–237, 10.1111/all.16404. 39673452 PMC11724241

[9] S. Narla , J. I. Silverberg , and E. L. Simpson , “Management of Inadequate Response and Adverse Effects to Dupilumab in Atopic Dermatitis,” Journal of the American Academy of Dermatology 86, no. 3 (2022): 628–636, 10.1016/j.jaad.2021.06.017. 34126094

[10] J. I. Silverberg , “Comorbidities and the Impact of Atopic Dermatitis,” Annals of Allergy, Asthma, & Immunology 123, no. 2 (2019): 144–151, 10.1016/j.anai.2019.04.020. 31034875

[11] R. S. Bumbacea , S. L. Corcea , S. Ali , L. Dinica , I. Fanfaret , and D. Boda , “Mite Allergy and Atopic Dermatitis: Is There a Clear Link? (Review),” Experimental and Therapeutic Medicine 20, no. 4 (2020): 3554–3560, 10.3892/etm.2020.9120. 32905207 PMC7465295

[12] J. Kim , S. Lee , S. Y. Woo , et al., “The Indoor Level of House Dust Mite Allergen Is Associated With Severity of Atopic Dermatitis in Children,” Journal of Korean Medical Science 28, no. 1 (2013): 74–79, 10.3346/jkms.2013.28.1.74. 23341715 PMC3546108

[13] S. R. Durham and M. H. Shamji , “Allergen Immunotherapy: Past, Present and Future,” Nature Reviews Immunology 23, no. 5 (2023): 317–328, 10.1038/s41577-022-00786-1. PMC9575636 36253555

[14] J. J. Yepes‐Nuñez , G. H. Guyatt , L. G. Gómez‐Escobar , et al., “Allergen Immunotherapy for Atopic Dermatitis: Systematic Review and Meta‐Analysis of Benefits and Harms,” Journal of Allergy and Clinical Immunology 151, no. 1 (2023): 147–158, 10.1016/j.jaci.2022.09.020. 36191689

[15] L. Yang and R. Zhu , “Immunotherapy of House Dust Mite Allergy,” Human Vaccines & Immunotherapeutics 13, no. 10 (2017): 2390–2396, 10.1080/21645515.2017.1364823. 28853977 PMC5647988

[16] M. Alvaro‐Lozano , C. A. Akdis , M. Akdis , et al., “EAACI Allergen Immunotherapy User’s Guide,” supplement, Pediatric Allergy & Immunology 31, no. S25 (2020): 1–101, 10.1111/pai.13189. 32436290 PMC7317851

[17] I. Agache , S. Lau , C. A. Akdis , et al., “EAACI Guidelines on Allergen Immunotherapy: House Dust Mite‐Driven Allergic Asthma,” Allergy 74, no. 5 (2019): 855–873, 10.1111/all.13749. 31095767

[18] J. M. Bae , Y. Y. Choi , C. O. Park , K. Y. Chung , and K. H. Lee , “Efficacy of Allergen‐Specific Immunotherapy for Atopic Dermatitis: A Systematic Review and Meta‐Analysis of Randomized Controlled Trials,” Journal of Allergy and Clinical Immunology 132, no. 1 (2013): 110–117, 10.1016/j.jaci.2013.02.044. 23647790

[19] H. Tam , M. A. Calderon , L. Manikam , et al., “Specific Allergen Immunotherapy for the Treatment of Atopic Eczema,” Cochrane Database of Systematic Reviews 2, no. 2 (2016): Cd008774, 10.1002/14651858.CD008774.pub2. 26871981 PMC8761476

[20] I. Agache , Y. Song , M. Posso , et al., “Efficacy and Safety of Dupilumab for Moderate‐to‐Severe Atopic Dermatitis: A Systematic Review for the EAACI Biologicals Guidelines,” Allergy 76, no. 1 (2021): 45–58, 10.1111/all.14510. 32691892

[21] F. Koskeridis , E. Evangelou , E. E. Ntzani , K. Kostikas , and S. Tsabouri , “Treatment With Dupilumab in Patients With Atopic Dermatitis: Systematic Review and Meta‐Analysis,” Journal of Cutaneous Medicine and Surgery 26, no. 6 (2022): 613–621, 10.1177/12034754221130969. 36214355

[22] J. I. Silverberg , J. P. Thyssen , K. Fahrbach , et al., “Comparative Efficacy and Safety of Systemic Therapies Used in Moderate‐to‐Severe Atopic Dermatitis: A Systematic Literature Review and Network Meta‐Analysis,” Journal of the European Academy of Dermatology and Venereology 35, no. 9 (2021): 1797–1810, 10.1111/jdv.17351. 33991374 PMC8453983

[23] M. J. Page , J. E. Mckenzie , P. M. Bossuyt , et al., “The PRISMA 2020 Statement: An Updated Guideline for Reporting Systematic Reviews,” BMJ 372 (2021): n71, 10.1136/bmj.n71. 33782057 PMC8005924

[24] D. Drevon , S. R. Fursa , and A. L. Malcolm , “Intercoder Reliability and Validity of WebPlotDigitizer in Extracting Graphed Data,” Behavior Modification 41, no. 2 (2017): 323–339, 10.1177/0145445516673998. 27760807

[25] X. Wan , W. Wang , J. Liu , and T. Tong , “Estimating the Sample Mean and Standard Deviation From the Sample Size, Median, Range and/or Interquartile Range,” BMC Medical Research Methodology 14, no. 1 (2014): 135, 10.1186/1471-2288-14-135. 25524443 PMC4383202

[26] G. Guyatt , Y. Wang , P. Eachempati , et al., “Core GRADE 4: Rating Certainty of evidence‐risk of Bias, Publication Bias, and Reasons for Rating up Certainty,” BMJ 389 (2025): e083864, 10.1136/bmj-2024-083864. 40360206

[27] C. A. Cuello‐Garcia , N. Santesso , R. L. Morgan , et al., “GRADE Guidance 24 Optimizing the Integration of Randomized and Non‐Randomized Studies of Interventions in Evidence Syntheses and Health Guidelines,” Journal of Clinical Epidemiology 142 (2022): 200–208, 10.1016/j.jclinepi.2021.11.026. 34800676 PMC8982640

[28] A. Bogacz‐Piaseczyńska , A. Bożek , M. Krupka‐Olek , A. Kawczyk‐Krupka , J. Zalejska‐Fiolka , and G. W. Canonica , “Dupilumab and House Dust Mite Immunotherapy in Patients With Atopic Dermatitis: A Preliminary Study,” Vaccines (Basel) 12, no. 9 (2024): 1046, 10.3390/vaccines12091046. 39340076 PMC11435717

[29] J. Kim , J. Boo , H. Jang , et al., “Combined Dupilumab and Allergen‐Specific Immunotherapy in Severe Refractory Atopic Dermatitis,” Allergy, Asthma & Immunology Research 16, no. 6 (2024): 682–689, 10.4168/aair.2024.16.6.682. PMC11621481 39622691

[30] J. Liu , L. Yang , Q. Xu , et al., “Allergen Immunotherapy and Dupilumab in Atopic Dermatitis: Clinical Efficacy and Disparities in Immunological Indicators,” World Allergy Organization Journal 18, no. 3 (2025): 101043, 10.1016/j.waojou.2025.101043. 40151542 PMC11946805

[31] Q. Q. Ye , Y. Liang , Y. Y. Li , et al., “Combined Dupilumab With Immunotherapy Is More Effective Than Dupilumab Alone in Children With Moderate‐to‐Severe Atopic Dermatitis,” International Immunopharmacology 163 (2025): 115143, 10.1016/j.intimp.2025.115143. 40695149

[32] C. K. Lo , D. Mertz , and M. Loeb , “Newcastle‐Ottawa Scale: Comparing Reviewers’ to Authors’ Assessments,” BMC Medical Research Methodology 14, no. 1 (2014): 45, 10.1186/1471-2288-14-45. 24690082 PMC4021422

[33] J. A. C. Sterne , J. Savović , M. J. Page , et al., “RoB 2: A Revised Tool for Assessing Risk of Bias in Randomised Trials,” BMJ 366 (2019): l4898, 10.1136/bmj.l4898. 31462531

[34] M. J. Gooderham , H. C. Hong , P. Eshtiaghi , and K. A. Papp , “Dupilumab: A Review of Its Use in the Treatment of Atopic Dermatitis,” supplement, Journal of the American Academy of Dermatology 78, no. 3 S1 (2018): S28–s36, 10.1016/j.jaad.2017.12.022. 29471919

[35] L. A. Beck , D. ThaçI , J. D. Hamilton , et al., “Dupilumab Treatment in Adults With Moderate‐to‐Severe Atopic Dermatitis,” New England Journal of Medicine 371, no. 2 (2014): 130–139, 10.1056/nejmoa1314768. 25006719

[36] C. A. Akdis and M. Akdis , “Mechanisms of Allergen‐Specific Immunotherapy and Immune Tolerance to Allergens,” World Allergy Organization Journal 8, no. 1 (2015): 17, 10.1186/s40413-015-0063-2. 26023323 PMC4430874

[37] M. Jutel , I. Agache , S. Bonini , et al., “International Consensus on Allergen Immunotherapy II: Mechanisms, Standardization, and Pharmacoeconomics,” Journal of Allergy and Clinical Immunology 137, no. 2 (2016): 358–368, 10.1016/j.jaci.2015.12.1300. 26853128

[38] M. Penagos and S. R. Durham , “Allergen Immunotherapy for Long‐Term Tolerance and Prevention,” Journal of Allergy and Clinical Immunology 149, no. 3 (2022): 802–811, 10.1016/j.jaci.2022.01.007. 35085663

[39] L. K. James , M. H. Shamji , S. M. Walker , et al., “Long‐Term Tolerance After Allergen Immunotherapy Is Accompanied by Selective Persistence of Blocking Antibodies,” Journal of Allergy and Clinical Immunology 127, no. 2 (2011): 509–16.e1‐5, 10.1016/j.jaci.2010.12.1080. 21281875

[40] L. Hesse , U. Brouwer , A. H. Petersen , et al., “Subcutaneous Immunotherapy Suppresses Th2 Inflammation and Induces Neutralizing Antibodies, but Sublingual Immunotherapy Suppresses Airway Hyperresponsiveness in Grass Pollen Mouse Models for Allergic Asthma,” Clinical and Experimental Allergy 48, no. 8 (2018): 1035–1049, 10.1111/cea.13169. 29752757

[41] C. James and D. I. Bernstein , “Allergen Immunotherapy: An Updated Review of Safety,” Current Opinion in Allergy and Clinical Immunology 17, no. 1 (2017): 55–59, 10.1097/aci.0000000000000335. 27906697 PMC5644500

[42] H. Y. Lee , S. M. Lee , S. Y. Kang , et al., “KAAACI Guidelines for Allergen Immunotherapy,” Allergy, Asthma & Immunology Research 15, no. 6 (2023): 725–756, 10.4168/aair.2023.15.6.725. PMC10643862 37957792

[43] B. Akinlade , E. Guttman‐Yassky , M. de Bruin‐Weller , et al., “Conjunctivitis in Dupilumab Clinical Trials,” British Journal of Dermatology 181, no. 3 (2019): 459–473, 10.1111/bjd.18276. 30851191 PMC6850316

[44] N. Neagu , C. Dianzani , G. Avallone , et al., “Dupilumab Ocular Side Effects in Patients With Atopic Dermatitis: A Systematic Review,” Journal of the European Academy of Dermatology and Venereology 36, no. 6 (2022): 820–835, 10.1111/jdv.17981. 35122335

