# Supplementary material for: Dupilumab Combined With House Dust Mite Allergen Immunotherapy for Atopic Dermatitis: A Systematic Review and Meta‐Analysis
Source: Clin Transl Allergy. 2026 Aug 8;16(8):e70194. doi: 10.1002/clt2.70194 (PMC13452629; doi:10.1002/clt2.70194)
Supplement: Supplementary file 1 [file CLT2-16-e70194-s001.docx]

eTable 1. Search strategy

| Database | Search strategy |
| --- | --- |
| PubMed | #1 "Dermatitis, Atopic"[Mesh]  OR atopic dermatitis[Title/Abstract]  OR atopic eczema[Title/Abstract]  OR eczema[Title/Abstract]  #2 "Dupilumab"[Mesh]  OR dupilumab[Title/Abstract]  #3 "Immunotherapy"[Mesh] OR "Desensitization, Immunologic"[Mesh] OR allergen immunotherapy[Title/Abstract] OR allergen-specific immunotherapy[Title/Abstract] OR specific immunotherapy[Title/Abstract] OR allergen specific immunotherapy[Title/Abstract] OR desensitization[Title/Abstract] OR hyposensitization[Title/Abstract] OR subcutaneous immunotherapy[Title/Abstract] OR sublingual immunotherapy[Title/Abstract] OR SCIT[Title/Abstract] OR SLIT[Title/Abstract] OR AIT[Title/Abstract]  #4 #1 AND #2 AND #3 |
| Embase | #1 'atopic dermatitis'/exp  OR 'atopic dermatitis':ti,ab,kw  OR 'atopic eczema':ti,ab,kw  OR eczema:ti,ab,kw  #2 'dupilumab'/exp  OR dupilumab:ti,ab,kw  #3 'allergen immunotherapy'/exp OR 'specific immunotherapy':ti,ab,kw OR 'allergen immunotherapy':ti,ab,kw OR 'allergen-specific immunotherapy':ti,ab,kw OR 'desensitization':ti,ab,kw OR 'hyposensitization':ti,ab,kw OR 'subcutaneous immunotherapy':ti,ab,kw OR 'sublingual immunotherapy':ti,ab,kw OR SCIT:ti,ab,kw OR SLIT:ti,ab,kw OR AIT:ti,ab,kw  #4 #1 AND #2 AND #3 |
| Web of Science | TS=(("atopic dermatitis" OR "atopic eczema" OR eczema) AND (dupilumab) AND ( "allergen immunotherapy" OR "allergen-specific immunotherapy" OR "specific immunotherapy" OR desensitization OR hyposensitization OR "subcutaneous immunotherapy" OR "sublingual immunotherapy" OR SCIT OR SLIT OR AIT)) |
| Cochrane Library | #1 MeSH descriptor: [Dermatitis, Atopic] explode all trees  #2 atopic dermatitis:ti,ab,kw OR atopic eczema:ti,ab,kw OR eczema:ti,ab,kw  #3 #1 OR #2  #4 MeSH descriptor: [Dupilumab] explode all trees  #5 dupilumab:ti,ab,kw  #6 #4 OR #5  #7 MeSH descriptor: [Immunotherapy] explode all trees  #8 MeSH descriptor: [Desensitization, Immunologic] explode all trees  #9 allergen immunotherapy:ti,ab,kw OR allergen-specific immunotherapy:ti,ab,kw OR specific immunotherapy:ti,ab,kw OR subcutaneous immunotherapy:ti,ab,kw OR sublingual immunotherapy:ti,ab,kw OR SCIT:ti,ab,kw OR SLIT:ti,ab,kw OR AIT:ti,ab,kw  #10 #7 OR #8 OR #9  #11 #3 AND #6 AND #10 |

eTable 2. Quality assessment using Newcastle-Ottawa Scale (NOS) for observational studies.

| Study | Selection | Comparability | Outcome/Exposure | Total Score |
| --- | --- | --- | --- | --- |
| Kim,2024 | ★★★★ | ★★ | ★★★ | 9 |
| Liu,2025 | ★★★★ | ★★ | ★★★ | 9 |
| Ye,2025 | ★★★★ | ★ | ★★★ | 8 |

eFigure 1. Quality assessment of RCT by Bogacz-Piaseczyńska,2024 using ROB2.

eFigure 2. Quality assessment of RCT by Bogacz-Piaseczyńska,2024 using ROB2.

As percentage (intention-to-treat)

|  | Low risk | Some concerns | High risk |
| --- | --- | --- | --- |
| Randomization process | 100 | 0 | 0 |
| Deviations from intended interventions | 0 | 100 | 0 |
| Mising outcome data | 100 | 0 | 0 |
| Measurement of the outcome | 100 | 0 | 0 |
| Selection of the reported result | 100 | 0 | 0 |
| Overall Bias | 0 | 100 | 0 |

eTable 3: Sensitivity analysis (leave-one-out)

| Outcome | Study,year | Treatment Effect | Tau-squared | I² | p-value |
| --- | --- | --- | --- | --- | --- |
| Change in disease severity at 6 months | Kim,2024 | -0.17(-0.62,0.27) | 0.00 | 0% | 0.45 |
|  | Liu,2025 | 0.09(-0.37,0.55) | 0.00 | 1% | 0.71 |
|  | Ye,2025 | 0.04(-0.62,0.70) | 0.09 | 40% | 0.91 |
| Change in disease severity at 12 months | Bogacz-Piaseczyńska,2024 | 1.03(-1.27,3.33) | 2.61 | 95% | 0.38 |
|  | Kim,2024 | 0.88(-1.72,3.47) | 3.37 | 96% | 0.51 |
|  | Ye,2025 | -0.30(-0.81,0.21) | 0.00 | 0% | 0.25 |
| Any adverse events | Bogacz-Piaseczyńska,2024 | 1.11(0.44,2.81) | 0.46 | 71% | 0.82 |
|  | Kim,2024 | 1.99(0.47,8.35) | 1.19 | 75% | 0.35 |
|  | Liu,2025 | 1.17(0.41,3.38) | 0.66 | 78% | 0.77 |
|  | Ye,2025 | 2.43(0.21,28.77) | 4.45 | 95% | 0.48 |
| Eye symptoms | Bogacz-Piaseczyńska,2024 | 0.77(0.34,1.73) | 0.00 | 0% | 0.52 |
|  | Kim,2024 | 0.65(0.24,1.75) | 0.00 | 0% | 0.39 |
|  | Liu,2025 | 0.71(0.31,1.65) | 0.00 | 0% | 0.43 |
|  | Ye,2025 | 0.56(0.20,1.62) | 0.00 | 0% | 0.29 |

eTable 4: GRADE summary of findings table.

| **Certainty assessment** | | | | | | | **№ of patients** | | **Effect** | | **Certainty** | **Importance** |
| --- | --- | --- | --- | --- | --- | --- | --- | --- | --- | --- | --- | --- |
| **№ of studies** | **Study design** | **Risk of bias** | **Inconsistency** | **Indirectness** | **Imprecision** | **Other considerations** | **Dupilumab +** **HDM-AIT** | **Dupilumab** | **Relative (95% CI)** | **Absolute (95% CI)** |  |  |
| **Disease severity at 6 months** | | | | | | | | | | | | |
| 3 | observational studies | not serious | not serious | not serious | serious^a^ | Nil | 52 | 54 | - | SMD 0.02 lower (0.41 lower to 0.36 higher) | ⨁◯◯◯ Very low ^a^ | CRITICAL |
| **Disease severity at 12 months** | | | | | | | | | | | | |
| 3 | 1 randomized trial and 2 observational studies | serious ^b^ | very serious ^c^ | not serious | serious ^d^ | Nil | 54 | 52 | - | SMD 0.53 higher (95% CI 1.10 lower to 2.16 higher) | ⨁◯◯◯ Very low ^b,c,d^ | CRITICAL |
| **Disease severity at 18 months** | | | | | | | | | | | | |
| 2 | observational studies | not serious | serious ^e^ | not serious | serious ^f^ | Nil | 36 | 38 | - | SMD 0.96 lower (95% CI 1.76 lower to 0.17 lower) | ⨁◯◯◯ Very low ^e,f^ | CRITICAL |
| **Disease severity at ≥ 30 months** | | | | | | | | | | | | |
| 2 | observational studies | not serious | very serious ^g^ | not serious | very serious ^h^ | Nil | 36 | 38 | - | SMD 1.16 lower (95% CI 2.56 lower to 0.24 higher) | ⨁◯◯◯ Very low ^g,h^ | CRITICAL |
| **Occurrence of ocular symptoms** | | | | | | | | | | | | |
| 4 | 1 randomized trial and 3 observational studies | serious ^i^ | not serious | not serious | serious ^j^ | Nil | 8/70 (11.4%) | 13/68 (19.1%) | RR 0.69 (0.31 to 1.51) | 59 fewer per 1,000 (from 132 fewer to 97 more) | ⨁◯◯◯ Very low ^i,j^ | CRITICAL |
| **Occurrence of any adverse events** | | | | | | | | | | | | |
| 4 | 1 randomized trial and 3 observational studies | serious ^k^ | very serious ^l^ | not serious | very serious ^m^ | Nil | 36/70 (51.4%) | 26/68 (38.2%) | RR 1.58 (0.46 to 5.40) | 222 more per 1,000 (from 206 fewer to 1,000 more) | ⨁◯◯◯ Very low ^k,l,m^ | CRITICAL |
| **DLQI scores at 6 months** | | | | | | | | | | | | |
| 2 | observational studies | not serious | not serious | not serious | Serious ^n^ | Nil | 38 | 40 | - | MD 1.40 higher (0.00 to 2.81 higher) | ⨁◯◯◯ Very low ^n^ | CRITICAL |
| **DLQI scores at 12 months** | | | | | | | | | | | | |
| 2 | 1 randomized trial and 1 observational study | serious ^o^ | very serious ^p^ | not serious | very serious ^q^ | Nil | 40 | 38 | - | MD 1.18 higher (2.67 lower to 5.03 higher) | ⨁◯◯◯ Very low ^o,p,q^ | CRITICAL |

**CI:** Confidence interval; **MD:** Mean difference; **RR:** Risk ratio; **SMD:** Standardised mean difference

#### Explanations

a. Downgraded for imprecision because of the small total sample size and the 95% confidence interval crossing the line of no effect, ranging from a possible slight benefit favoring combination therapy to a possible slight benefit favoring monotherapy.

b. Downgraded by one level for risk of bias because the evidence included two observational cohort studies with potential residual confounding and one randomized trial rated as having some concerns in the RoB 2 assessment.

c. Downgraded by two levels for very serious inconsistency because substantial heterogeneity was observed across studies (I² = 93%), with marked differences in both the magnitude and direction of effect estimates.

d. Downgraded by one level for serious imprecision because the total sample size was small and the 95% confidence interval was very wide, crossing the line of no effect and including both a possible benefit favoring combination therapy and a possible benefit favoring monotherapy.

e. Downgraded by one level for inconsistency because moderate heterogeneity was observed (I² = 61%), although the direction of effect was consistent across studies.

f. Downgraded by one level for imprecision because the total sample size was small and the confidence interval was wide, despite not crossing the line of no effect.

g. Downgraded by two levels for very serious inconsistency because substantial heterogeneity was observed (I² = 87%), with marked differences in the magnitude of effect estimates between studies.

h. Downgraded by two levels for very serious imprecision because the total sample size was small and the 95% confidence interval was very wide, crossing the line of no effect and ranging from a possible large benefit favoring combination therapy to no clear difference.

i. Downgraded by one level for risk of bias because the evidence included three observational cohort studies with potential residual confounding and one randomized trial rated as having some concerns in the RoB 2 assessment.

j. Downgraded by one level for imprecision because the number of events was limited and the 95% confidence interval crossed the line of no effect, including both a possible reduction and a possible increase in ocular symptoms.

k. Downgraded by one level for risk of bias because the evidence included three observational cohort studies with potential residual confounding and one randomized trial rated as having some concerns in the RoB 2 assessment. In addition, adverse event reporting may have varied across studies.

l. Downgraded by two levels for very serious inconsistency because substantial heterogeneity was observed across studies (I² = 86%), with marked differences in the magnitude and direction of effect estimates.

m. Downgraded by two levels for very serious imprecision because the confidence interval was very wide and crossed the line of no effect, including both a possible reduction and a possible substantial increase in adverse events.

n. Downgraded by one level for imprecision because the total sample size was small and the confidence interval included no clear difference between groups.

o. Downgraded by one level for risk of bias because the evidence included one observational cohort study with potential residual confounding and one randomized trial rated as having some concerns in the RoB 2 assessment.

p. Downgraded by two levels for very serious inconsistency because substantial heterogeneity was observed across studies (I² = 96%), with opposite directions of effect estimates.

q. Downgraded by two levels for very serious imprecision because the total sample size was small and the 95% confidence interval was very wide, crossing the line of no effect and including both a possible benefit favoring combination therapy and a possible benefit favoring monotherapy.
